# Evaluation of Anomalous Coronary Arteries from the Pulmonary Artery

**DOI:** 10.21470/1678-9741-2016-0082

**Published:** 2017

**Authors:** Alper Guzeltas, Erkut Ozturk, Ibrahim Cansaran Tanidir, Taner Kasar, Sertac Haydin

**Affiliations:** 1 Department of Pediatric Cardiology, Istanbul Saglik Bilimleri University, Mehmet Akif Ersoy Thoracic and Cardiovascular Surgery Center, Istanbul, Turkey.; 2 Department of Pediatric Cardiovascular Surgery, Istanbul Saglik Bilimleri University, Mehmet Akif Ersoy Thoracic and Cardiovascular Surgery Center, Istanbul, Turkey.

**Keywords:** Coronary Vessel Anomalies, Bland White Garland Syndrome, Pulmonary Artery/Abnormalities, Cardiac Surgical Procedures

## Abstract

**Objective:**

This study evaluated clinical and diagnostic findings, treatment methods, and follow-up of cases of anomalous coronary arteries from the pulmonary artery.

**Methods:**

The study included all cases diagnosed with anomalous coronary arteries from the pulmonary artery between January 2012 and January 2016. Data from patients’ demographic characteristics, electrocardiography, echocardiography, angiographic findings, operation, intensive care unit stay, and follow-up were evaluated.

**Results:**

The study included 12 patients (8 male, 4 female), 10 with anomalous left coronary artery from the pulmonary artery (ALCAPA) and 2 with anomalous right coronary artery from the pulmonary artery (ARCAPA). Median age at diagnosis was 4 months (range, 1 month - 10 years old) and median weight was 5.5 kg (range, 3-30 kg). The most common complaints were murmur (n=7) and respiratory distress (n=5). In 4 cases, the initial diagnosis was dilated cardiomyopathy. Electrocardiographs were pathologic in all cases. Echocardiographic examination revealed medium to severe mitral valve regurgitation in 4 cases and reduced (< 40%) ejection fraction in 6 patients. Of the 12 patients, 8 underwent direct implantation of the left coronary artery into the aorta, 2 underwent implantation of the right coronary artery into the aorta, and the remaining 2 underwent a Takeuchi procedure. There were no early mortalities. Median hospital stay was 20 days (range, 5-35 days). Median follow-up duration was 18 months (range, 5-36 months), and no cases required further surgery during follow-up.

**Conclusions:**

Anomalous coronary arteries from the pulmonary artery can be successfully repaired providing there is early diagnosis and effective, appropriate intensive care unit follow-up. Therefore, coronary artery origins should be evaluated carefully, especially in cases with dilated cardiomyopathies.

**Table:** 

Abbreviations, acronyms & symbols				
ALCAPA ARCAPA CVP ECG ECHO ECMO EF	= Anomalous left coronary artery from the pulmonary artery = Anomalous right coronary artery from the pulmonary artery = Central venous pressure = Electrocardiography = Echocardiography = Extracorporeal membrane oxygenation = Ejection fraction		ICU LCAs LV NIRS PICU RCA SD TPN	= Intensive care unit = Left coronary arteries = Left ventricular = Cranial near infrared spectroscopy = Pediatric intensive care unit = Right coronary artery = Standard deviation = Total parenteral nutrition

## INTRODUCTION

Anomalous coronary arteries from the pulmonary artery are among the least-common congenital heart diseases. The majority of cases are of an anomalous left coronary artery from the pulmonary artery (ALCAPA), while cases of an anomalous right coronary artery from the pulmonary artery (ARCAPA) are rarely seen^[1,2]^.

The findings and timeline for symptom development may vary depending on the effectiveness of the myocardial collateral branches concurrent with pulmonary artery pressure decrease. Some patients may be completely asymptomatic whereas others may experience death in the early period due to heart failure, valvular insufficiencies or myocardial infarction^[1,2]^.

The definitive treatment is surgery to normalize myocardial perfusion, after which, progressive remodeling and improvement in left ventricular (LV) function may be seen. Depending on the preference of the cardiac unit, either direct anastomosis may be performed on the anomalous coronary artery from the pulmonary artery to the aorta or, for patients in whom direct transfer of the coronary artery is not feasible, an intrapulmonary aortocoronary tunnel, as described by Takeuchi, may be implemented successfully^[3-6]^.

This study evaluated presentation findings, diagnosis, treatment methods, and follow-up results of patients at a single tertiary center who were diagnosed with anomalous coronary arteries from the pulmonary artery.

## METHODS

### Study Design

For this retrospective study, the center’s computer database was searched for all patients diagnosed with anomalous coronary arteries from the pulmonary artery between January 1, 2012 and January 1, 2016. Cases with additional congenital heart diseases were excluded.

Patients’ demographic data, including age, gender, presentation type, initial diagnosis, and electrocardiography (ECG), telecardiography, echocardiography (ECHO), cardiac catheterization, and angiographic findings were evaluated. In addition, surgical methods, intensive care unit (ICU) follow-up data, observed complications, and outpatient-clinic follow-up results were assessed.

ECGs were evaluated according to the literature for sinus tachycardia, presence of pathological Q wave, ST elevation, ST depression, etc. ECHO findings before surgery, during ICU follow-up, and after hospital discharge were compared. Two-dimensional, M-mode, and Doppler ECHOs were performed using standard imaging techniques, in accordance with the recommendations of the American Society of Echocardiography.

Surgical techniques, namely the Takeuchi procedure and coronary implantation, were applied in accordance with the literature.

### Postoperative Care

Patients were transferred to the ICU intubated, and mechanical ventilator support was initiated. In some patients, sternal closure was delayed.

In the ICU, all patients were monitored non-invasively by pulse oximetry, ECG, end-tidal capnography, and cranial near infrared spectroscopy (NIRS) and invasively by arterial blood pressure, central venous pressure (CVP), and left atrial pressure. Milrinone (0.5 microgram/kg/min) and low doses of epinephrine (0.05 microgram/kg/min) were the preferred inotropic support, beginning in the first postoperative hours. Noradrenaline support was added if necessary. Fentanyl and midazolam were used for sedation and analgesia. All patients were started on total parenteral nutrition (TPN) in addition to minimal enteral feeding within the first postoperative day.

Excessive volume loading was avoided. Vasodilator therapy, core cooling, neuromuscular blocking, and peritoneal dialysis were initiated in cases of low cardiac output that did not respond to inotropic support. Daily postoperative ECHO was performed in the pediatric intensive care unit (PICU). Tapering of postoperative medication, including inotropes, and weaning from the mechanical ventilator were decided on case by case, based on clinical evaluation and LV function on ECHO.

### Statistical Analysis

Statistical analysis of the data was performed using SPSS for Windows, version 15.0 (SPSS Inc.; Chicago, IL, USA). Categorical variables were presented as absolute and percent frequencies, and quantitative variables were summarized as means and standard deviation (SD).

## RESULTS

### Demographic and Clinical Findings

This retrospective study included 12 patients, 10 with ALCAPA and 2 with ARCAPA. There were 8 males and 4 females. Median age at diagnosis was 4 months (range, 1 month to 10 years old), and median weight was 5.5 kg (range, 3-30 kg). The most common complaints were murmur (n=7) and respiratory distress (n=5). In 4 cases, the initial diagnosis was dilated cardiomyopathy. Table 1 shows the patients’ demographic data.

**Table 1:** Demographic and clinical findings of patients.

Patient Number	Sex/age (months)	Symptoms	ECG	Echocardiography	Catheter Angiography
1*	F/20	Murmur	Sinus tachycardia	EF = 30% LVEDd Z score: + 2 MR = mild IC = + PH = + RCA/AA = > 1.4	ALCAPA
2*	M/120	Murmur	V5-V6 T negativity	EF = 55% LVEDd Z score: 0-1 MR = severe IC = + PH = - RCA/AA = >1.4	ALCAPA
3*	M/8	Shortness of breath, DCM	ALMI	EF = 30% LVEDd Z score = > +3 MR = severe IC = + PH = + RCA/AA > 1.4	ALCAPA
4*	M/4	Restlessness, murmur	ALMI	EF = 40% LVEDd Z score = > + 2 MR = mild IC = + PH = + RCA/AA= >1.4	ALCAPA
5*	F/3	Shortness of breath, DCM	ALMI	EF = 35-40% LVEDd Z score = > + 3 MR = severe IC = + PH = + RCA/AA = >1.4	ALCAPA
6*	F/2	Shortness of breath, DCM	ALMI	EF: 25% LVEDd Z score = > +4 MR = mild IC = + PH = + RCA/AA = >1.4	ALCAPA
7*	M/8	Murmur	V5-V6 T negativity	EF: 30% LVEDd Z score = > +3 MR = mild IC = + PH = + RCA/AA = >1.4	ALCAPA
8*	M/4	Shortness of breath, DCM	ALMI	EF: 25% LVEDd Z score = >+4 MR = mild IC = + PH = + RCA/AA = >1.4	ALCAPA
9*	M/1	Restlessness, Murmur	Sinus tachycardiaALMI	EF = 45% LVEDd Z score = > +2 MR = moderate IC = + PH = + RCA/AA = >1.4	ALCAPA
10*	M/4	Shortness of breath	ALMI	EF = 40% LVEDd Z score = > +2 MR = mild IC = + PH = + RCA/AA = > 1.4	ALCAPA
11 &	F/1	Murmur	Sinus tachycardiaV1-V3 T negativity	EF = 60 % LVEDd Z score = 0-1 MR = none IC = + PH = - LCA/AA = >1.3	ARCAPA
12 &	M/18	Murmur	V1-V3 T negativity	EF = 70 % LVEDd Z score = 0-1 MR = none IC = + PH = - LCA/AA = > 1.3	ARCAPA

* ALCAPA=anomalous left coronary artery from the pulmonary artery; &ARCAPA=anomalous right coronary artery from the pulmonary artery; ALMI=anterolateral myocardial infarction; DCM=dilated cardiomyopathy; ECG=electrocardiography; EF=ejection fraction; F=female; IC=collaterals; LCA/AA=left coronary artery/aorta artery; LVEDd= Left Ventricular End Diastolic Diameter; M=male; MR=mitral regurgitation; PA=pulmonary artery; PH=papillary hyperechogenicity; Pt=patient; RCA=right coronary artery

### Electrocardiographic and Echocardiographic Findings

**Fig. 1:**
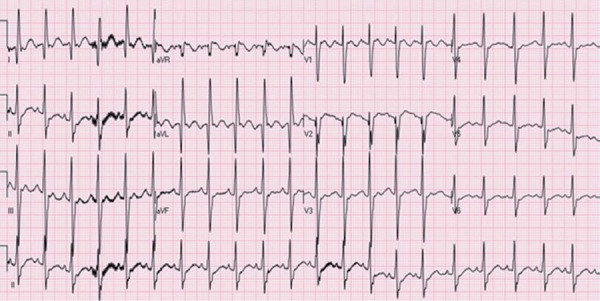
Preoperative twelve lead electrocardiogram of patient 9. Electrocardiogram shows signs of acute anterolateral myocardial infarction; deep Q waves, ST segment elevation and T-wave inversion in leads I and aVL.

The ECGs of 7 patients showed a deep Q wave in the DI, aVL, and V6 derivations. It revealed sinus tachycardia in 5 cases, T wave inversion in the V5‒V6 derivation in 2 cases, and T wave inversion in the V1‒V3 derivation in 2 cases (Figure 1).

**Fig. 2:**
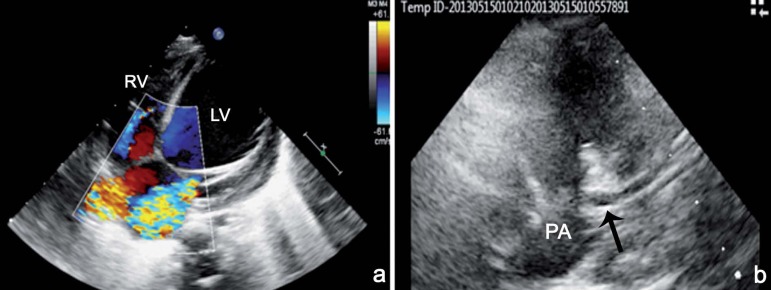
A - Apical four chamber view echocardiogram showing dilated left ventricle, hyperechogenicity in papillary muscles, ventricular septaldefect-like appearance due to coronary collaterals and significant mitral regurgitation; B - Parasternal short axis view echocardiogram showing anomalous origin of the left coronary artery from pulmonary artery.

Of the 10 patients with ALCAPA, 9 had varying degrees of LV dilatation and dysfunction. ECHO revealed varying degrees of mitral valve regurgitation (Figure 2) and collateral blood flow on the interventricular septum in all cases, papillary muscle hyperechogenicity in 9 cases, dilated right coronary artery (RCA) in 8 cases, and retrograde diastolic flow toward the pulmonary artery in 4 cases. At the initial ECHO examination, coronary arteries of 4 patients were erroneously thought to originate from the aorta.

In the 2 patients with ARCAPA, LV systolic and diastolic diameters were within the normal limits. In both cases, the left coronary arteries (LCAs) were dilated secondarily. One patient with ARCAPA had had previous ECHO and catheter angiography, which had diagnosed coronary-cameral fistula.

### Catheterization and Angiography

**Fig. 3:**
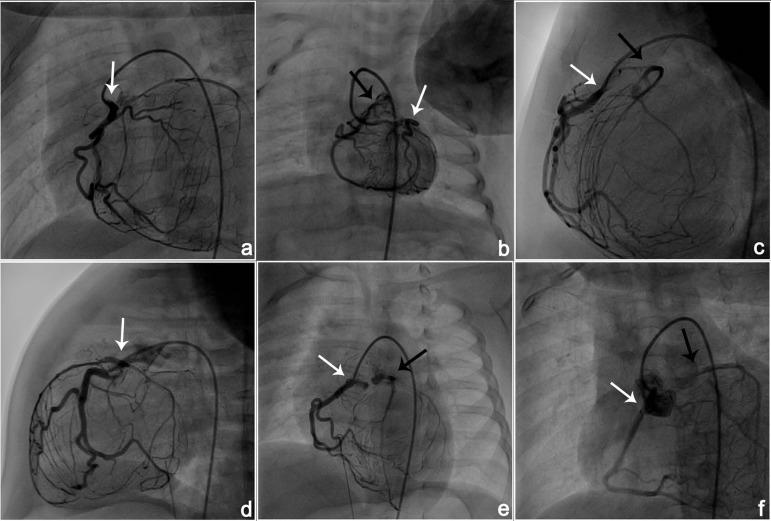
A, B - Angiographic image of ARCAPA; white arrows showing left coronary artery and black arrows showing right coronary artery and C-F - Angiographic image of ALCAPA; white arrows showing right coronary artery and black arrows showing left coronary artery.”

All patients were evaluated with cardiac catheterization and angiography (Figure 3).

### Surgical Treatment

Among the 10 patients with ALCAPA, 8 underwent direct implantation of the LCA into the aorta and 2 underwent the Takeuchi technique. In the 2 patients with ARCAPA, coronary reimplantation was applied. Median surgery time was 140 minutes (range, 76-375 minutes). In 3 cases, closure of the sternum was delayed and, in those cases, mean closure time was 96 hours.

There were no early mortalities. Ventricular tachycardia occurred in 4 cases. In 3 cases, blood cultures were positive. In addition, 2 patients needed high-dose inotrope support, and another 2 patients needed peritoneal dialysis. Mean duration of mechanical ventilation was 54 hours (range, 12-360 hours), mean length of ICU stay was 9 days (range, 2-10 days), and mean length of hospital stay was 20 days (range, 5-35 days). Extracorporeal membrane oxygenation (ECMO) support was used for 1 patient. Table 2 shows surgical, ICU-stay, and follow-up data.

**Table 2:** Patients intensive care unit and follow-up data.

Patient	Operation	OperationTime (min)	Sternum closed	PICU complications	MV	ICU	Hospital length of stay	Follow-up data
1 *	LMCA pericardial hood augmentation & reimplantation	260	Closed	None	12	3	10	Discharged 26-month follow-up EF = 60% MR = mild PS = 20 mmHg
2 *	LMCA pericardial hood augmentation & reimplantation & no mitral valve ring implantation	375	Closed	Ventricular tachycardia,Peritoneal dialysis requirement	20	5	8	Discharged 34-month follow-up EF = 45% MR = medium
3 *	Takeuchi operation	160	Open/5 days	High dose inotrope requirement,Peritoneal dialysis requirement	120	19	26	Discharged 20-month follow-up EF = 50% PS = 40mmHg MR = mild AR = mild
4 *	Takeuchi operation	180	Closed	Ventricular tachycardia	72	12	20	Discharged 16-month follow-up EF = 60%
5 *	Direct reimplantation of LMCA to the ascending aorta	178	Closed	High dose inotrope requirement,Ventricular tachycardia	240	19	24	Discharged 30-month follow-up EF = 55% MR = mild AR = mild
6 *	Direct reimplantation of LMCA to the ascending aorta	152	Open/2 days	High dose inotrope requirement,Positive blood culture for *Klebsiella pneumoniae*	360	22	35	Discharged 24-month follow-up EF = 70%
7 *	Direct reimplantation of LMCA to the ascending aorta	123	Closed	Ventricular tachycardia,Positive blood culture for *Klebsiella pneumoniae*	120	16	24	Discharged12-month follow-upEF = 65-70%,MR = mild
8 *	Direct reimplantation of LMCA to the ascending aorta	125	Closed	None	36	6	20	Discharged 22-month follow-up EF = 50-55% MR = mild LV aneurysm
9 *	Direct reimplantation of LMCA to the ascending aorta	94	Open/5 days	High dose inotrope requirement,Positive blood culture for *S. Epidermis* and *Pseudomonas*	284	17	25	Discharged 7-month follow-up EF = 60% MR = mild Apical hypokinesiaIncreased LV trabeculation
10 *	Direct reimplantation of LMCA to the ascending aorta	105	Closed	None	24	3	8	Discharged 5-month follow-up EF = 50%MR = trivial
11 &	RCA ostium transfer to the aorta	91	Closed	None	12	3	7	Discharged 15-month follow-up No problem
12 &	RCA ostium transfer to the aorta	76	Closed	None	16	2	5	Discharged 7-month follow-up No problem

* ALCAPA=anomalous left coronary artery from the pulmonary artery; & ARCAPA=anomalous right coronary artery from the pulmonary artery; AR=aortic regurgitation; EF=ejection fraction; ICU=intensive care unit; LMCA=left main coronary artery; LV=left ventricle; MR= mitral regurgitation; MV=mechanical ventilation; PICU=pediatric intensive care unit; PS=pulmonary stenosis; RCA=right coronary artery;

### Follow-Up

Median follow-up duration was 18 months (range, 5‒34 months). No patients required further surgery. Two patients (1 Takeuchi, 1 coronary reimplantation) developed mild pulmonary stenosis, and one patient (Takeuchi) had mild aortic regurgitation, developed during follow-up. The most recent ECHO evaluation showed that the ejection fraction of every patient was over 45%. Mild mitral valve regurgitation was observed in 5 patients and moderate mitral valve regurgitation in 1 patient (Table 2).

## DISCUSSION

ALCAPA is a rare disorder, affecting 1 in 300,000 live births and representing approximately 0.5% of congenital heart defects^[6,7]^. Presentation and diagnosis timelines for ALCAPA vary depending on pulmonary vascular resistance and the presence of collateral vessels between the right and left coronary artery systems. A wide range of symptoms has been reported in the literature, including an one month-old baby with heart failure symptoms and an asymptomatic 53-year-old who was diagnosed incidentally^[8]^. A Danish study of 9 patients with ALCAPA reported that 77% had heart failure symptoms, including shortness of breath and wheezing, while the remaining 23% were asymptomatic at presentation^[9]^. A Chinese study of 27 cases reported acute heart failure (n=15), pneumonia (n=7), and cardiac murmurs (n=5) as presenting symptoms^[10]^. In the present study, 60% of patients presented with murmur, 40% with shortness of breath, and 30% were asymptomatic.

Because myocardial ischemia develops over time in an anterolateral distribution, pathological Q waves can be seen in derivations representing the region, namely the DI, aVL, and V4-V6. In patients with extensive collateral circulation, there might be nonspecific ECG changes^[5]^. In patients with impaired LV functions, typical ECG changes should indicate the presence of an anomalous coronary artery^[5,6]^.

Rodriguez-Gonzalez et al.^[1]^ conducted a study of 12 patients and reported that 6 had specific ECG changes compatible with acute lateral myocardial infarction. In the present study, the ECGs of 7 patients revealed pathological Q waves in the DI, aVL, and V6. The ECGs of the other 5 patients showed nonspecific changes.

ECHO findings included the following: dilatation of the coronary artery with normal origin, increased ratio of the diameter of the coronary artery originating from the normal sinus to the aortic annulus (> 0.14), increased echogenicity of the papillary muscles, increased flow toward the pulmonary artery, and either identification of an inappropriate origin of the anomalous coronary artery from the pulmonary artery or non-identification of the origin of the coronary artery. However, initial ECHOs have shown that 50-70% of patients had their coronary arteries arising from the aorta^[7,11]^. In the present study, 4 patients with ALCAPA and 1 with ARCAPA were diagnosed with normal coronary-artery patterns, and the ratio of false negative misdiagnoses was 43%.

Anomalous coronary arteries from the pulmonary artery might be falsely diagnosed as idiopathic dilated cardiomyopathy or endocardial fibroelastosis. Zheng et al.^[5]^ reported that 18 of 21 cases (78%) in their study were initially misdiagnosed. In the present study, 4 patients had initially been diagnosed with idiopathic dilated cardiomyopathy.

Various surgical methods can be used to repair an anomalous coronary artery from the pulmonary artery, all of them aiming to establish a system with two coronary arteries. One of the most common methods is direct reimplantation of the coronary artery into the aorta. The other is creating an aortopulmonary window that directs blood flow from the aorta to the LCA (Takeuchi). After those procedures, no matter how impaired the ventricular functions had been, myocardial function can quickly heal^[12,13]^.

Jin et al.^[14]^ reported that 11 patients treated by direct reimplantation experienced improved LV function. In addition, Sarioglu et al.^[3]^ reported the recovery of LV function in all patients, except for 1 mortality (5 coronary reimplantations, 2 Takeuchi). Ayik et al.^[15]^ reported that 10 patients treated by the Takeuchi method had a 10% mortality rate. In the present study, coronary reimplantation was applied in 8 patients and the Takeuchi method in 2 patients. Regardless of the technique applied, LV function became normal in all patients.

In patients with ALCAPA, LV function is characterized by mitral valve dysfunction. Preoperative mitral valve regurgitation is a significant risk factor. However, mitral valve repair is controversial in ALCAPA patients. Vouhé et al.^[16]^ suggested that resolution of myocardial ischemia leads to improved papillary muscle function. Therefore, they proposed that mitral valve repair should not be done at first. In patients with ongoing mitral valve regurgitation, coronary artery restenosis should be investigated and, if necessary, the mitral valve should be repaired at this stage. In contrast, other researchers have argued that simultaneous mitral valve repair and anterolateral commissural annuloplasty during coronary artery reimplantation enhance early recovery of LV functions^[17]^. In the present study, only 1 patient needed mitral valve repair during the first surgery. Mitral valve regurgitation improved in all patients except one, who required mitral valve repair during initial surgery.

ARCAPA, a rare coronary anomaly, was first described in 1885. It represents approximately 0.002% of all congenital heart diseases. Unlike ALCAPA, it is often asymptomatic during infancy and early childhood. During adolescence and adulthood, presentation often consists of a murmur and it rarely includes chest pain, heart failure, arrhythmia, or sudden death. Time to diagnosis shows a wide distribution, from 1 month old to 90 years old. There is no consensus in the literature regarding treatment methods and diagnosis times in ARCAPA patients^[18]^.

Although there are reports of various treatment methods, including surveillance, medical therapy, surgery, and surgical ligation, due to the 10-18% risk of sudden death, most authors advocate creating a dual coronary circulation by direct reimplantation, even in asymptomatic patients^[18,19]^. In the present study, both of the ARCAPA patients were asymptomatic, but underwent surgery, one at 9 months old and the other at 18 months old, in accordance with the literature.

Reported mortality rates range from 0-16%^[20-22]^. The literature suggests that the main reasons for mortality are low cardiac output and ventricular arrhythmias, and it advises using advanced life-support systems to reduce mortality. In the present study, 3 patients developed low cardiac output and 4 developed ventricular tachycardia. No ECMO was required, nor was there any early mortality.

### Limitations

The most important limitations of the present study were the small number of patients included and its retrospective nature. In addition, the follow-up period was relatively short compared with those in the literature.

## CONCLUSION

Anomalous coronary arteries from the pulmonary artery may present with various clinical, ECG, and ECHO findings. The condition can be successfully treated by surgery if accompanied by early diagnosis and effective, appropriate ICU follow-up. Coronary artery origins should be carefully evaluated, especially in patients with dilated cardiomyopathy.

**Table:** 

Authors’ roles & responsibilities	
AG	Conception and study design; analysis and/or data interpretation; statistical analysis; final manuscript approval
EO	Conception and study design; analysis and/or data interpretation; statistical analysis; final manuscript approval
ICT	Conception and study design; analysis and/or data interpretation; statistical analysis; final manuscript approval
TK	Conception and study design; analysis and/or data interpretation; statistical analysis; final manuscript approval
SH	Execution of operations and/or trials; manuscript writing or critical review of its content; final manuscript approval
